# Correction: Five-year outcomes of photodynamic therapy combined with intravitreal injection of ranibizumab or aflibercept for polypoidal choroidal vasculopathy

**DOI:** 10.1371/journal.pone.0230505

**Published:** 2020-03-11

**Authors:** Wataru Kikushima, Atsushi Sugiyama, Seigo Yoneyama, Mio Matsubara, Yoshiko Fukuda, Ravi Parikh, Yoichi Sakurada

The first author’s first and last name appear in the incorrect order. The correct name is: Wataru Kikushima. The correct citation is: Kikushima W, Sugiyama A, Yoneyama S, Matsubara M, Fukuda Y, Parikh R, et al. (2020) Five-year outcomes of photodynamic therapy combined with intravitreal injection of ranibizumab or aflibercept for polypoidal choroidal vasculopathy. PLoS ONE 15(2): e0229231. https://doi.org/10.1371/journal.pone.0229231
